# Delirium and Functional Recovery in Patients Discharged to Skilled Nursing Facilities After Hospitalization for Heart Failure

**DOI:** 10.1001/jamanetworkopen.2020.37968

**Published:** 2021-03-16

**Authors:** Caroline Madrigal, Jenny Kim, Lan Jiang, Jacob Lafo, Melanie Bozzay, Jennifer Primack, Stephen Correia, Sebhat Erqou, Wen-Chih Wu, James L. Rudolph

**Affiliations:** 1 Providence VA Medical Center, Center of Innovation in Long Term Services and Supports, Providence, Rhode Island; 2 Department of Medicine, Warren Alpert Medical School of Brown University, Providence, Rhode Island; 3 Department of Psychiatry & Human Behavior, Brown University, Providence, Rhode Island; 4 Providence VA Medical Center, Center for Neurorestoration and Neurotechnology, Providence, Rhode Island; 5 Butler Hospital, Providence, Rhode Island; 6 Center for Gerontology, Brown University School of Public Health, Providence, Rhode Island

## Abstract

**Importance:**

A substantial number of patients discharged to skilled nursing facilities (SNFs) after heart failure (HF) hospitalization experience regression in function or do not improve. Delirium is one of few modifiable risk factors in this patient population. Therefore, understanding the role of delirium in functional recovery may be useful for improving outcomes.

**Objective:**

To assess the association of delirium with 30-day functional improvement in patients discharged to SNFs after HF hospitalization.

**Design, Setting, and Participants:**

This retrospective cohort study included patients hospitalized for HF in 129 US Department of Veterans Affairs hospitals who were discharged to SNFs from October 1, 2010, to September 30, 2015. Data were analyzed from June 14 to December 18, 2020.

**Exposures:**

Delirium, as determined by the Minimum Data Set (MDS) 3.0 Confusion Assessment Method, with dementia as a covariate, determined via *International Classification of Diseases, Ninth Revision (ICD-9)* coding.

**Main Outcomes and Measures:**

The difference between admission and 30-day MDS 3.0 Activities of Daily Living (ADL) scores.

**Results:**

A total of 20 495 patients (mean [SD] age, 78 [10.3] years; 78.9% White; and 97% male) were included in the analysis. Of the total sample, 882 patients (4.3%) had delirium on an SNF admission. The mean (SD) baseline ADL score on admission to SNF was significantly worse among patients with delirium than without (18.3 [4.7] vs 16.1 [5.2]; *P* < .001; *d* = 0.44.). On the 30-day repeated assessment, mean (SD) function (ADL scores) improved for both patients with delirium (0.6 [2.9]) and without delirium (1.8 [3.6]) (*P* < .001; *d* = −0.38). In the multivariate adjusted model, delirium was associated with statistically significant lower ADL improvement (difference in ADL score, –1.07; 95% CI, –1.31 to –0.83; *P* < .001).

**Conclusions and Relevance:**

In this retrospective cohort study, patients with HF discharged to SNFs with delirium were less likely to show improvement in function compared with patients without delirium. Findings suggest a potential need to reexamine how and when health care professionals assess delirium in HF patients throughout their hospitalization and SNF course. Identifying and treating delirium for HF patients earlier in their care trajectory may play an important role in improving care and long-term functional outcomes in this population. Future research is warranted to further investigate the association between delirium and functional recovery for HF and other patient populations.

## Key Points

**Question:**

Is delirium associated with 30-day functional recovery in patients discharged to skilled nursing facilities after heart failure hospitalization?

**Findings:**

In this cohort study of 20 495 patients from the US Department of Veterans Affairs health system discharged to skilled nursing facilities after heart failure hospitalization, delirium was associated with poor functional recovery. The baseline Activities of Daily Living score on admission to a skilled nursing facility was significantly worse among patients with delirium; patients with delirium also had significantly lower Activities of Daily Living improvement from baseline to follow-up assessments.

**Meaning:**

Study results suggest that delirium on admission to skilled nursing facilities after heart failure hospitalization was associated with poor functional recovery.

## Teaser

This cohort study assesses the association between delirium and functional recovery for patients recently discharged to skilled nursing facilities after heart failure hospitalization.

## Introduction

Heart failure (HF) is a chronic, functionally disabling condition projected to affect more than 8 million US residents by 2030.^1^ Acute exacerbations of HF are a leading cause of hospitalizations and rehospitalizations.^2^ Although many patients return home after hospitalization, almost 20% of patients with HF are unable to function independently and are consequently discharged to skilled nursing facilities (SNFs) to facilitate functional recovery.^3^ Functional recovery after HF hospitalization is a substantial factor to facilitate independence, quality of life, and survival.^4^ Therefore, it is advisable to identify and minimize factors that negatively affect functional recovery for HF patients discharged to SNFs.^5^

Delirium is an acute reversible change in mental status characterized by fluctuations in awareness and attention, which often result in complications, such as rehospitalization and delayed discharge from SNFs.^6,7^ Although delirium is modifiable and potentially preventable, it is common in patients with HF and associated with poor outcomes.^8^ In contrast, cognitive impairment is a common chronic condition among patients with HF, owing in part to cerebral hypoperfusion among other pathophysiological mechanisms.^9^ There is a growing body of literature on the co-occurrence of delirium and dementia and the predisposing factors and repercussions of co-occurrence.^10,11^ However, to our knowledge, the differential associations between delirium and dementia and functional recovery have not been well characterized,^8,12^ and it is unclear whether delirium affects functional recovery for those with HF independent of dementia.^13^

A better understanding of the association between delirium and functional recovery may have potential implications for improving outcomes for patients with HF. In this study, we explore the association between delirium and functional recovery for patients recently discharged to SNFs after HF hospitalization. We hypothesized that delirium at SNF admission would be independently associated with worse functional recovery.

## Methods

The study was approved by the institutional review board of the Providence US Department of Veterans Affairs (VA) Medical Center, and informed consent was waived because there was minimal risk posed to patients. This study followed the Strengthening the Reporting of Observational Studies in Epidemiology (STROBE) reporting guideline for cohort studies.^14^

### Sample

Veterans Health Administration electronic records were used to retrospectively identify veterans from 129 VA Medical Centers hospitalized with a primary diagnosis of HF and discharged to community-based skilled nursing facilities (SNFs) outside the VA health system between October 1, 2010, and September 30, 2015. A total of 673 101 veterans with unique HF admissions were identified in the Veterans Health Administration records. After applying our exclusion criteria, the final sample consisted of 20 495 veterans (eFigure 1 in the Supplement). Veterans were excluded from the study if they received palliative or hospice care services before, during, or at hospital discharge or if the only follow-up Minimum Data Set (MDS) 3.0 assessment occurred after the 90-day assessment interval because the scope of this study focused on short-term functional recovery. Veterans were also excluded if they did not have complete data (eg, 2 or more MDS assessments).

### Delirium

The MDS 3.0 is a federally mandated standardized assessment administered to residents within 14 days of admission to a certified SNF and every 30 days thereafter or at discharge from the SNF. Presence or absence of delirium at SNF admission was classified using the MDS 3.0 Confusion Assessment Method (CAM). The Confusion Assessment Method^15,16^ is a widely used highly reliable diagnostic algorithm for the identification of delirium. The Confusion Assessment Method accounts for the following cardinal features of delirium: (1) acute onset and fluctuating course, (2) inattention, (3) disorganized thinking, and (4) altered level of consciousness.^17^ Based on CAM score, veterans were classified as having or not having delirium. Delirium was categorized as (1) presence of acute onset or fluctuating course and (2) inattention, coupled with (3) disorganized thinking or (4) altered level of consciousness. This method has been previously described^18^ as an inclusive dichotomization of CAM because feature 1 is joined by *or* rather than *and*. This dichotomization is important owing to delirium being potentially underreported using the MDS 3.0 version of CAM.^19^

### Function

The MDS 3.0 Activities of Daily Living (ADL) assessment includes self-reported data to assess a resident’s ability to function within an SNF on 10 activities including bed mobility, transfer, walk in room, walk in corridor, locomotion on unit, locomotion off unit, dressing, eating, toilet use, and personal hygiene. Each ADL is scored on a numeric scale from 0 to 4, with higher scores representing increased dependence: independent (ADL = 0), needs supervision (ADL = 1), needs limited assistance (ADL = 2), needs extensive assistance (ADL = 3), or total dependence (ADL = 4); scores of 7 or 8 (activity occurred only once or twice or activity did not occur) were recoded to 4, consistent with previous research.^18,19^ Total scores ranged from 0 to 28 (with higher scores indicating lower functional ability). Functional recovery was calculated by subtracting ADL scores at 30 days from admission scores.^19^ The primary outcome was the difference between admission and 30-day MDS 3.0 Activities of Daily Living (ADL) scores.

Our primary outcome of interest was functional recovery. We calculated functional recovery by subtracting total ADL scores at 30 days from total ADL scores at admission. If the 30-day ADL assessment was not present, it was replaced using assessments with proximity to the 30-day ADL assessment as the primary end point. The hierarchy of ADL assessments for replacing the 30-day ADL assessment was 15-day, 45-day, 60-day, then 90-day. If no additional assessments were available, the veteran was excluded from the cohort (eFigure 1 in the Supplement). For communicating clinical relevance, the continuous functional recovery scores were categorized into 3 groups: functional improvement (scores >0), no change in function (scores=0), and functional decline (scores <0).

### Additional Variables

Dementia was a covariate in this study because delirium and dementia often coexist in hospitalized patients.^20^ Preexisting dementia was found using inpatient and outpatient coding via the *International Classification of Diseases, Ninth Revision (ICD-9)* from the Centers for Medicare & Medicaid Chronic Condition Warehouse at any point before SNF admission. eTable 1 in the Supplement lists the codes used. Veterans Health Administration electronic health records were used to find demographic information, including age, sex, and race/ethnicity. The Elixhauser comorbidity index was used to identify comorbidities via *ICD-9* codes drawn from VA data in the year before admission. The VA health care costs in the year before admission were obtained from VA accounting records. Use of VA resources (eg, number of emergency department visits, length of stay of hospitalization immediately preceding SNF placement) was collected from VA administrative records. Ejection fraction (%) was gathered from natural language processing of echocardiography reports in the year prior^21^ and was categorized with clinical convention (ie, reduced, borderline, or preserved).^22^

### Statistical Analysis

The baseline characteristics of those with delirium were compared with those without delirium using *t* tests for continuous variables and χ^2^ tests for noncontinuous categorical variables. Standardized mean differences (Cohen *d*) were calculated as the difference in means divided by the SD of the entire sample.^23^ Standardized mean differences help to contextualize the size of associations between variables and groups (eg, veterans with or without delirium); effect sizes can be interpreted as small (0.2), medium (0.5), or large (0.8).^24^ A linear mixed-effects model was used to model the difference in ADL from baseline assessment to the post 30-day assessment among the 2 groups. The adjusted model included covariates of age, sex, race/ethnicity, dementia, Elixhauser comorbidity index, emergency department admissions, ejection fraction category, length of hospital stay, cost of health care use, and baseline ADL scores. All covariates were treated as fixed effects.

The VA Medical Centers facility was included as a random effect to account for potential clustering within the facility. Our modeling used the exchangeable correlation matrix, in which the diagonal elements represent the variance and off-diagonal elements represent covariance or correlation. The 95% CIs were calculated for the linear mixed-effects models. Output from the modeling is displayed as the regression β coefficient, which can be interpreted as the mean ADL change from baseline over time between those with and without delirium, adjusted for the covariates of interest specified above. Data were analyzed from June 14 to December 18, 2020. A 2-sided *P* < .05 was the threshold for statistical significance. Statistical modeling was performed using SAS statistical software, version 9.4 (SAS Institute Inc).

## Results

### Population Characteristics

The cohort consisted of 20 495 veterans (mean [SD] age, 78 [10.3] years; 78.9% White; and 97% male) discharged from VA hospitals to SNFs after HF hospitalization. Baseline characteristics of participants are reported in Table 1. Veterans had a substantial comorbidity burden: 32% (n = 6606) had a diagnosis of dementia before HF admission, 51% had diabetes, 43% had chronic lung disease, 24% had depression, and 21% had obesity (Table 1).

**Table 1.** Comparison of Characteristics of SNF Residents With and Without Delirium After HF Hospitalization

Characteristic	Overall cohort (N = 20 495)	No delirium (n = 19 613)	Delirium (n = 882)	*P* value	SMD
Age, mean (SD)^a^, y	77.6 (10.3)	77.5 (10.3)	81.0 (9.3)	<.001	0.36
Female sex, No. (%)^a^	622 (3.0)	591 (3.0)	31 (3.5)	.40	0.03
Race/ethnicity, No. (%)^a^					
Missing	45 (0.2)	45 (0.2)	0		
White	16 167 (78.9)	15 457 (78.8)	710 (80.5)	.38	0.06
Black	3295 (16.1)	3157 (16.1)	138 (15.7)		
Hispanic	396 (1.9)	382 (2.0)	14 (1.6)		
Other^b^	592 (2.9)	572 (2.9)	20 (2.3)		
Dementia, No. (%)^a^	6606 (32.2)	6081 (31.0)	525 (59.5)	<.001	0.60
Elixhauser comorbidity index, mean (SD)^a^^,^^c^	4.8 (2.8)	4.8 (2.8)	4.3 (2.8)	<.001	–0.18
Comorbidities, No. (%)					
Chronic lung disease	8889 (43.4)	8572 (43.7)	317 (35.9)	<.001	–0.16
Diabetes	10 425 (50.9)	10 038 (51.2)	387 (43.9)	<.001	–0.15
Diabetes with complications	5822 (28.4)	5632 (28.7)	190 (21.5)	<.001	–0.17
Hypothyroidism	2999 (14.6)	2862 (14.6)	137 (15.5)	.44	0.03
Chronic kidney disease	1967 (9.6)	1903 (9.7)	64 (7.3)	.02	–0.09
Liver disease	1092 (5.3)	1058 (5.4)	34 (3.9)	.05	–0.07
Tumor history	3533 (17.2)	3405 (17.4)	128 (14.5)	.03	–0.08
Obesity	4280 (20.9)	4165 (21.2)	115 (13.0)	<.001	–0.22
Weight loss	1986 (9.7)	1888 (9.6)	98 (11.1)	.14	0.05
Anemia	7855 (38.3)	7550 (38.5)	305 (34.6)	.02	–0.08
Alcohol use disorder	1611 (7.9)	1547 (7.9)	64 (7.3)	.50	–0.02
Substance use disorder	793 (3.9)	770 (3.9)	23 (2.6)	.05	–0.07
Mental health	3269 (16.0)	3089 (15.8)	180 (20.4)	<.001	0.12
Depression	4913 (24.0)	4707 (24.0)	206 (23.4)	.66	–0.02
Emergency department admissions in year preceding hospitalization, mean (SD)^a^	3.1 (4.1)	3.1 (4.0)	2.7 (4.1)	.01	–0.09
Left ventricular ejection fraction in year preceding hospitalization (among nonmissing), mean (SD)	43.4 (14.9)	43.4 (14.9)	43.5 (14.6)	.94	0.00
Ejection fraction categories, No. (%)^a^					
0%-40%, Reduced	5255 (25.6)	5058 (25.8)	197 (22.3)	<.001	0.21
40%-50%, Borderline	3014 (14.7)	2891 (14.7)	123 (14.0)		
>50%, Preserved	5507 (26.9)	5312 (27.1)	195 (22.1)		
Missing	6719 (32.8)	6352 (32.4)	367 (41.6)		
Length of stay of HF hospitalization preceding SNF admission, mean (SD), d^a^	10.56 (9.27)	10.6 (9.3)	10.5 (9.0)	.88	–0.01
Health care use in year preceding hospitalization, mean (SD), $^a^	30 630 (38 450)	30 778 (38 556)	27 339 (35 860)	.01	–0.09

Abbreviations: HF, heart failure; SMD, standardized mean difference; SNF, skilled nursing facility.

^a^ Variable adjusted for in linear mixed-effects model.

^b^ Other includes those who identified themselves as American Indian or Alaska Native, Asian, Native Hawaiian or Other Pacific Islander.

^c^ Elixhauser comorbidity data were collected prehospitalization.

On admission assessment to SNFs, 882 veterans (4.3%) had delirium and 19 613 (95.7%) did not. Veterans with delirium were more likely to be older (mean [SD] age, 81.0 [9.3] vs 77.5 [10.3]; *P* < .001), with a history of dementia (59.5% vs 31.0%; *P* < .001) and lower comorbidity burden (Elixhauser comorbidity index, 4.3 vs 4.8) compared with those without delirium. The mean (SD) left ventricular ejection fraction was comparable between veterans with and without delirium (43.4 [14.9] vs 43.4 [14.9]; *P* = .94) as was length of hospital stay for HF admission (10.5 [9.0] vs 10.6 [9.3]; *P* = .88) (Table 1).

### Outcomes

Table 2 presents the baseline and follow-up ADL assessments at SNFs according to delirium classification. The mean (SD) baseline ADL score on admission to SNFs was significantly worse among those with delirium (18.3 [4.7] vs 16.1 [5.2]; *P* < .001, *d* = 0.44) than those without. Among the veterans with baseline delirium, 21.7% (n = 191) had worse functional performance, 41.3% (n = 364) showed no change, and 37.1% (n = 327) improved in functional performance between their initial baseline and follow-up ADL assessments (values given are for the 30-day assessment unless replaced with the 15-, 45-, 60-, or 90-day if no 30-day assessment available) (Table 2; eFigure 2 in the Supplement). In contrast, among the 19 613 veterans without delirium on admission to SNFs, 14.4% (n = 2821) had worse functional performance, 33.9% (n = 6655) showed no change, and 51.7% (n = 10 137) improved over the same time frame. Overall, veterans with delirium improved their ADL score by a mean (SD) of 0.6 (2.9) points from baseline to follow-up, but those without delirium had a significantly greater mean (SD) improvement of 1.8 (3.6) points (*P* < .001, *d* = −0.38).

**Table 2.** Overall ADL Outcomes in SNF Residents According to Delirium Classification

Variable	Overall	No delirium	Delirium	*P* value	SMD
Patients, No. (%)	20 495	19 613 (95.7)	882 (4.3)		
ADL score on SNF admission, mean (SD)^a^	16.2 (5.2)	16.1 (5.2)	18.3 (4.7)	<.001	0.44
ADL score change from admission to follow-up assessment, mean (SD)^b^	1.8 (3.6)	1.8 (3.6)	0.6 (2.9)	<.001	–0.38
Categorical ADL score change from admission to follow-up assessment, No. (%)^b^					
Worse functional performance	3012 (14.7)	2821(14.4)	191 (21.7)	<.001	0.32
No change	7019 (34.3)	6655 (33.9)	364 (41.3)		
Improved functional performance	10 464 (51.1)	10 137 (51.7)	327 (37.1)		

Abbreviations: ADL, Activities of Daily Living; SMD, standardized mean difference; SNF, skilled nursing facility.

^a^ Variable adjusted for in linear mixed-effects model.

^b^ Follow-up assessment was at 30 days unless it was unavailable then the 15-, 45-, 60-, or 90-day assessment was used.

In both the unadjusted and adjusted multivariate linear mixed-effects models (Table 3), those with delirium were significantly less likely to experience functional improvement between admission and follow-up assessment than those without delirium. In the unadjusted model, those with delirium had a mean change in ADL score of −1.23 (95% CI, –1.47 to –1.00; *P* < .001) compared with those without delirium. In the adjusted model, those with delirium had a mean change in ADL score of −1.07 (95% CI, –1.31 to –0.83; *P* < .001) compared with those without delirium. Preexisting diagnoses of dementia were associated with worse mean (SD) ADL scores at SNF admission after HF hospitalization (17.1 [5.0]; *d* = 0.27; *P* < .001) and worse functional recovery on follow-up assessment in SNFs (1.3 [3.2]; *d* = −0.21; *P* < .001) (eTable 2 in the Supplement).

**Table 3.** Unadjusted and Adjusted Linear Mixed-Effects Models for ADL Change

Model	Difference in ADL score change^a^ (95% CI)	*P* value
Unadjusted model		
Delirium	–1.23 (–1.47 to –1.00)	<.001
Adjusted models		
Delirium adjusted for age, sex, race/ethnicity, dementia, Elixhauser comorbidity index, emergency department admissions, ejection fraction category, length of hospital stay, cost of health care use, baseline ADL score, and random effect of facility	–1.07 (–1.31 to –0.83)	<.001

Abbreviation: ADL, activities of daily living.

^a^ Comparing delirium with no delirium. Patients with delirium improved by 1.07 points less in the ADL scale than those without delirium at 30 days.

## Discussion

This cohort study examined the association between delirium and functional recovery of veterans in SNFs after hospitalization because of HF. To our knowledge, this is the first study to assess the association between delirium and functional ability for the population with HF in SNFs. Baseline function (ADL score) in this study cohort was impaired, as may be expected in patients with HF who may require SNF admission, but those with delirium had worse baseline scores, were less likely to improve in function, and were more likely to show no change or experience regression in function compared with those without delirium.

Delirium is a reversible disturbance in mental status with fluctuations in awareness and attention, characteristics that have a negative association with patients’ functional abilities. However, despite delirium being a reversible condition, study results suggest that even in SNFs, facilities designed to promote functional recovery, patients with delirium were less likely to improve in function than patients without. Potential mechanisms driving this association include the likelihood that these patients are less likely to participate in self-care education, physical activities, and rehabilitation. The association between delirium and functional recovery for patients with HF may be a marker of slow long-term functional recovery, if any, which may hold decision-making implications for families and health system implications for insurance payers.

Patients with HF may be particularly vulnerable to experiencing delirium and negative functional outcomes. Atherosclerosis is not selective to the arteries of the heart—it affects all arteries, including those in the brain; therefore, HF may exacerbate cognitive changes that reduce functional recovery. Past research links brain atherosclerosis, particularly in the white matter, with cognitive changes and highlights associations between leukoaraiosis and delirium.^25,26,27^ Our finding that decreased functional recovery is associated with delirium is consistent with previous research, which found delirium to be associated with poor functional recovery in primarily operative settings.^28,29^ The risk of delirium specifically for patients with HF has been further explored by Parente et al,^30^ who found HF was an independent risk factor for postoperative delirium, and Mathillis et al,^31^ who found HF was an independent risk factor for the co-occurrence of delirium and dementia.

Our adjusted model demonstrated decreased improvement of 1 point (–1.07) on the MDS 3.0 ADL assessment for patients with delirium. It is important to note the clinical implications of a change of 1 point on the ADL assessment because it could be the difference between needing assistance from staff vs complete dependence on staff or total independence vs staff supervision. Each change in ADL score has potential repercussions on a patient’s perceived level of ability and engagement in care that could ultimately affect their likelihood of being discharged home, especially when coupled with cognitive challenges, such as delirium. Functional ability on the MDS 3.0 ADL assessment is also associated with the development of individualized care plans^32^; therefore the level of care deemed essential could affect many other clinical factors, such as level of assistance provided to patients and required staffing numbers and skill mix.^33^

The importance of categorizing a patient’s level of function is not limited to their time in the SNF. There is an opportunity to reconsider the assessment of delirium and function in the broader context of HF. For example, the New York Heart Association Classification^34^ is used to grade patients’ HF severity (Classes I-IV) via an assessment of symptoms associated with physical activity, but the scale focuses solely on physical symptoms that precipitate functional decline. There is an opportunity to consider the intersection of cognitive and physical symptoms when determining their association with function and HF severity. The ADL assessment used in this study is distinct from symptoms associated with functional limitation (eg, fatigue, palpitation, and dyspnea); however, research on the cross-validity of other functional measures on the MDS 3.0 (eg, Section GG) and functional assessments like the New York Heart Association Classification could serve as a first step toward incorporating mental status into existing measurements of functional ability after replication and expansion of this study’s findings.

Another approach to addressing the association between delirium and functional recovery may be to prioritize the early assessment of delirium in this patient group, perhaps before a patient’s discharge to an SNF so that acute and subacute reversible medical factors that could prolong the course of delirium can be corrected. However, delirium is frequently missed without a standardized assessment, and a standardized hospital assessment for delirium does not currently exist. Even with a standardized assessment like the MDS 3.0, the results of systemic screening may be less than ideal without clear leadership and maintenance.

The implementation and maintenance of delirium assessments and subsequent treatment may be warranted in the context of the association between brain and cardiac function. The systematic assessment of delirium during acute care hospitalization and before discharge may be important for maximizing functional recovery in patients with HF. Future studies on delirium assessment and treatment may have important implications for improving functional recovery in patients with HF. Future research may also prove essential in understanding the potential applicability of our findings on the association between delirium and decreased functional recovery in other patient populations.

### Strengths and Limitations

A strength of this study is its large racially and geographically diverse population. Like most VA data, the study sample is predominantly male, but this factor does not entirely limit the generalizability of results. Because no standardized assessments of cognitive or physical function are performed at hospitals, this study used the MDS 3.0 CAM assessment from SNFs. There are a few potential drawbacks of using the MDS 3.0 CAM. First, the assessment is administered within 14 days of SNF admission, which may limit our ability to assess delirium and immediate hospital discharge and may not account for cases of delirium that could have resolved between discharge and assessment, all of which could have underestimated the observed association. The MDS 3.0 CAM does not capture the duration of delirium, which is an important feature when considering functional recovery. While there is validation data on the MDS CAM,^18^ the pragmatic implementation of the MDS may be subject to the limitations of other reporting responsibilities, with variable motivation for completeness and accuracy. The administration of the MDS 3.0 CAM also does not align with the intended rigor of CAM administration,^20^ which likely resulted in mostly severe cases of delirium being detected in this study. Even with limitations associated with the MDS 3.0 CAM, our findings align with other postacute care studies on delirium facing similar limitations.^19,35,36^

Additional limitations of this work include our reliance on *ICD-9* coding to ascertain the presence of dementia, as it historically is not the most sensitive or comprehensive measure, which may have resulted in an underestimation of the prevalence of dementia in this population.^37,38^ While triangulation of *ICD* codes and data from electronical health records would be ideal,^39^ triangulation was not possible in this study. We included *ICD-9* codes from Medicare and VA data to maximize the likelihood of detecting dementia. In addition, this study does not explore the co-occurrence of delirium and dementia, which may be an important area for future research because of a growing understanding of the interplay between the 2 conditions.

## Conclusions

In this study, patients with HF discharged to SNFs who had delirium on admission were less likely to demonstrate functional improvement and more likely to experience regression in function compared with patients without delirium. This population may commonly have multiple comorbidities that put them at risk for reduced functional improvement, but the discovery of the association of delirium with function is notable because delirium is modifiable. Increased emphasis on identifying and treating delirium for patients with HF before discharge and throughout their SNF course may be important for functional recovery, but more research is needed. To improve functional outcomes of the rapidly growing HF population, future studies may wish to investigate the association between delirium treatment and change in functional recovery in patients with HF during their functional recovery posthospitalization.

## Notes

Supplement. **eTable 1.** ICD-9 Codes and Descriptions Used to Identify Dementia **eTable 2.** Overall Activities of Daily Living (ADL) Outcomes in Skilled Nursing Facility (SNF) Residents According to Dementia Classification **eFigure 1.** Cohort Flowchart **eFigure 2.** Overall Activities of Daily Living (ADL) Outcomes by Delirium Status
