# Supplementary material for: Delirium and Functional Recovery in Patients Discharged to Skilled Nursing Facilities After Hospitalization for Heart Failure
Source: JAMA Netw Open. 2021 Mar 16;4(3):e2037968. doi: 10.1001/jamanetworkopen.2020.37968 (PMC7967078; doi:10.1001/jamanetworkopen.2020.37968)
Supplement: Supplement. — eTable 1. ICD-9 Codes and Descriptions Used to Identify Dementia eTable 2. Overall Activities of Daily Living (ADL) Outcomes in Skilled Nursing Facility (SNF) Residents According to Dementia Classification eFigure 1. Cohort Flowchart eFigure 2. Overall Activities of Daily Living (ADL) Outcomes by Delirium Status [file jamanetwopen-e2037968-s001.pdf]

Supplemental Online Content

Madrigal C, Kim J, Jiang L, et al. Delirium and functional recovery in patients discharged to skilled nursing facilities after hospitalization for heart failure. *JAMA Netw Open*. 2021;4(3):e2037968. doi:10.1001/jamanetworkopen.2020.37968

eTable 1. ICD-9 Codes and Descriptions Used to Identify Dementia

eTable 2. Overall Activities of Daily Living (ADL) Outcomes in Skilled Nursing Facility (SNF) Residents According to Dementia Classification

eFigure 1. Cohort Flowchart

eFigure 2. Overall Activities of Daily Living (ADL) Outcomes by Delirium Status

This supplemental material has been provided by the authors to give readers additional information about their work.

© 2021 Madrigal C et al. *JAMA Network Open*.

eTable 1: ICD-9 Codes and Descriptions Used to Identify Dementia

ICD-9 Code	ICD-9 Description
046.11	Variant Creutzfeldt-Jakob disease
046.19	Other and unspecified Creutzfeldt-Jakob disease
046.3	Progressive multifocal leukoencephalopathy
046.71	Gerstmann-straussler-scheinker syndrome
046.79	Other and unspecified prion disease of central nervous system
046.9	Unspecified slow virus infection of central nervous system
290.0	Senile dementia, uncomplicated
290.10	Presenile dementia, uncomplicated
290.11	Presenile dementia with delirium
290.12	Presenile dementia with delusional features
290.13	Presenile dementia with depressive features
290.20	Senile dementia with delusional features
290.21	Senile dementia with depressive features
290.3	Senile dementia with delirium
290.40	Vascular dementia, uncomplicated
290.41	Vascular dementia, with delirium
290.42	Vascular dementia, with delusions
290.43	Vascular dementia, with depressed mood
291.1	Alcohol-induced persisting amnestic disorder
291.2	Alcohol-induced persisting dementia
292.82	Drug-induced persisting dementia
294.1	Dementia in conditions classified elsewhere
294.10	Dementia in conditions classified elsewhere without behavioral disturbance
294.11	Dementia in conditions classified elsewhere with behavioral disturbance
294.20	Dementia, unspecified, without behavioral disturbance
294.21	Dementia, unspecified, with behavioral disturbance
331.0	Alzheimer's disease
331.11	Pick's disease

© 2021 Madrigal C et al. *JAMA Network Open*.

ICD-9 Code	ICD-9 Description
331.19	Other frontotemporal dementia
331.82	Dementia with Lewy bodies
294.11/042 .0	Dementia in conditions classified elsewhere with behavioral disturbance/hiv with specified infections
294.10/042.0	Dementia in conditions classified elsewhere without behavioral disturbance/hiv with specified infections
294.11/331.5	Dementia in conditions classified elsewhere with behavioral disturbance/idiopathic normal pressure hydrocephalus (inph)
294.10/331.5	Dementia in conditions classified elsewhere without behavioral disturbance/idiopathic normal pressure hydrocephalus (inph)
294.11/332.0	Dementia in conditions classified elsewhere with behavioral disturbance/Parkinson's disease
294.10/332.0	Dementia in conditions classified elsewhere without behavioral disturbance/Parkinson's disease
294.11/333.4	Dementia in conditions classified elsewhere with behavioral disturbance/Huntington's chorea
294.10/333.4	Dementia in conditions classified elsewhere without behavioral disturbance/Huntington's chorea

© 2021 Madrigal C et al. *JAMA Network Open*.

eTable 2. Overall Activities of Daily Living (ADL) outcomes in skilled nursing facility (SNF) residents according to dementia classification

	No Dementia<br>N =13889	Dementia<br>N =6606	P-value	Standardized mean difference
ADL score upon SNF admission—mean (sd)	15.8 (5.2)	17.1 (5.0)	<.001	0.27
ADL score change from admission to follow-up assessment—mean (sd)	2.0 (3.7)	1.3 (3.2)	<.001	-0.21
Categorical ADL score change from admission to follow-up assessment—n (%)			<.001	0.21
Worse Functional Performance	1926 (13.9)	1086 (16.4)		
No Change	4591 (33.1)	2428 (36.8)		
Improved Functional Performance	7372 (53.1)	3092 (46.8)		

© 2021 Madrigal C et al. *JAMA Network Open*.

eFigure 1. Cohort Flowchart

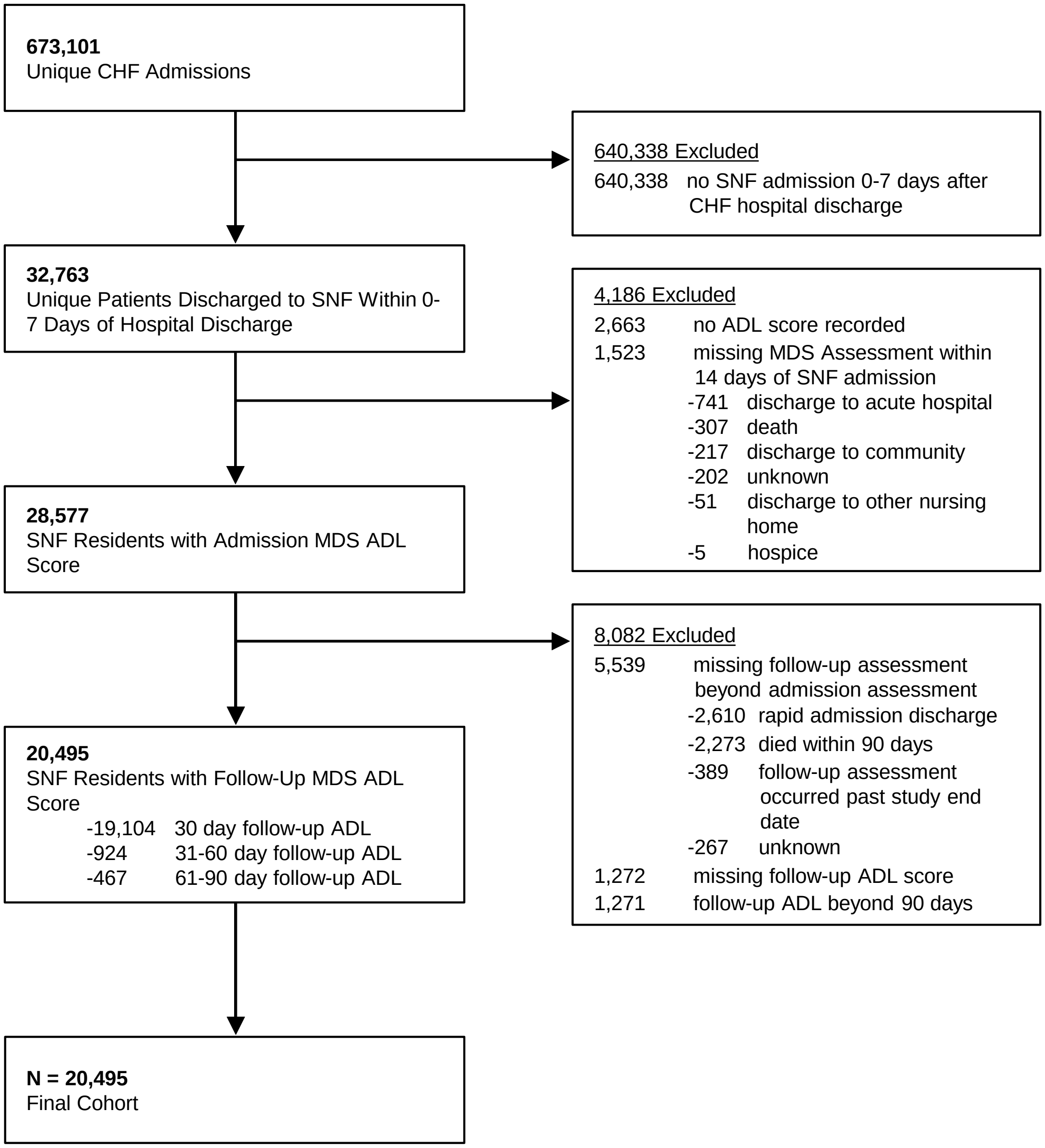

***From 673,101 unique admissions, we identified 20,495 with the desired exposure and outcomes. Exclusions were due to missing admission MDS data and missing 30-day MDS data.**

***ADL MDS - Activities of daily living MDS 3.0**

***SNF - Skilled Nursing Facility**

© 2021 Madrigal C et al. *JAMA Network Open*.

eFigure 2. Overall Activities of Daily Living (ADL) Outcomes by Delirium Status

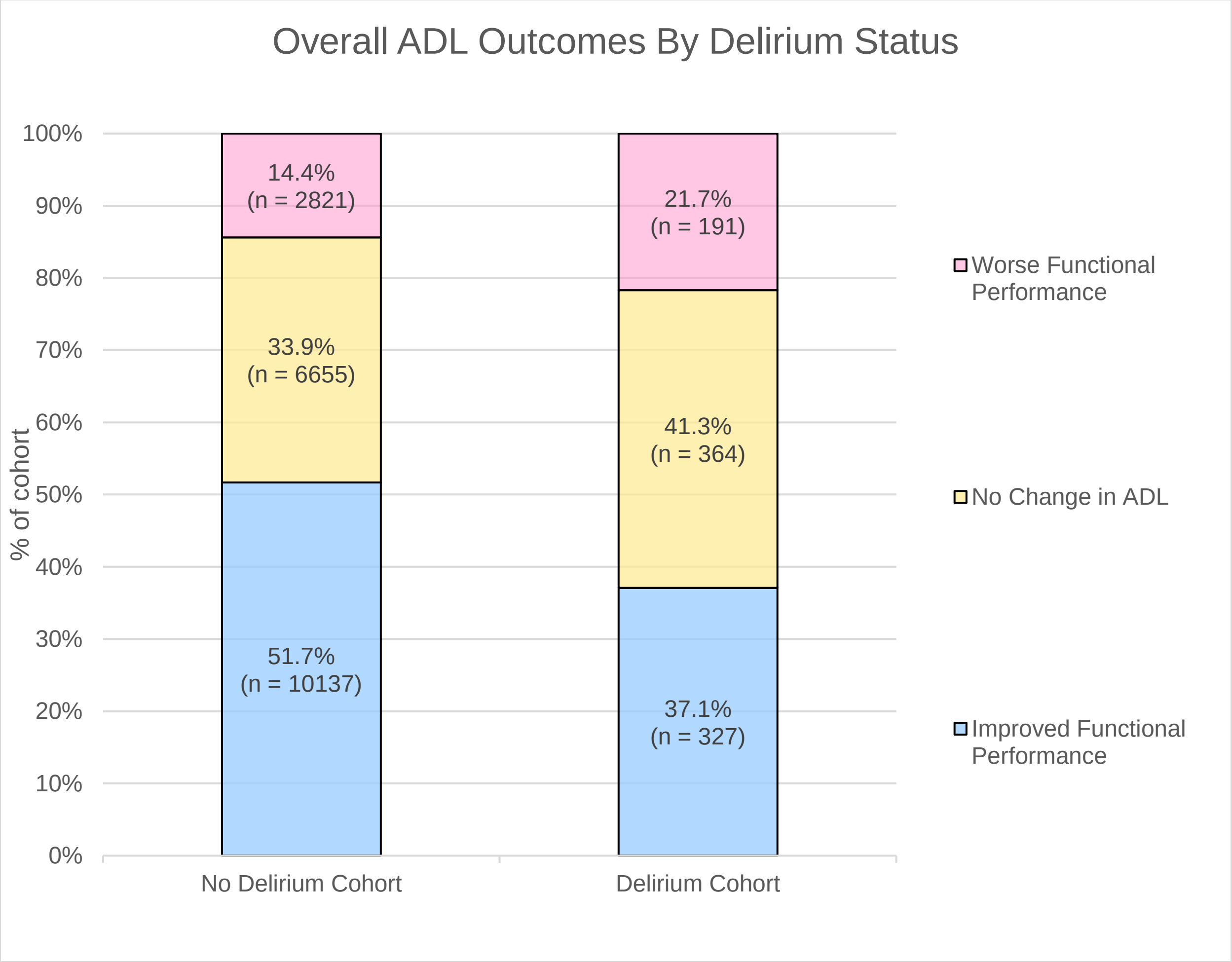

© 2021 Madrigal C et al. *JAMA Network Open*.
